# Characterizing HDAC Pathway Copy Number Variation in Pan-Cancer

**DOI:** 10.3389/pore.2022.1610288

**Published:** 2022-06-13

**Authors:** Shuming Yang, Shengzhi Xie, Xinying Shi, Dan Su, Bo He, Yang Xu, Zhefeng Liu

**Affiliations:** ^1^ Department of Oncology, Senior Department of Oncology, The Third Medical Center of PLA General Hospital, Beijing, China; ^2^ Department of Oncology, Senior Department of Oncology, The Fifth Medical Center of PLA General Hospital, Beijing, China; ^3^ Genecast Biotechnology Co., Ltd., Wuxi, China

**Keywords:** prognosis, copy number variation, immune, histone deacetylase, tumor characteristics

## Abstract

**Background:** Histone deacetylase (HDAC) plays a crucial role in regulating the expression and activity of a variety of genes associated with tumor progression and immunotherapeutic processes. The aim of this study was to characterize HDAC pathway copy number variation (CNV) in pan-cancer.

**Methods:** A total of 10,678 tumor samples involving 33 types of tumors from The Cancer Genome Atlas (TCGA) were included in the study.

**Results:** HDAC pathway CNV and CNV gain were identified as prognostic risk factors for pan-cancer species. The differences of tumor characteristics including tumor mutational burden, tumor neoantigen burden, high-microsatellite instability, and microsatellite stable between HDAC pathway CNV altered-type group and wild-type group varied among the various cancer species. In some cancer types, HDAC pathway CNV alteration was positively correlated with loss of heterozygosity, CNV burden, ploidy, and homologous recombination defect score markers, while it was significantly negatively correlated with immune score and stroma score. There were significant differences in immune characteristics such as major histocompatibility complex class I (MHC-I), MHC-II, chemokines, cytolytic-activity, and IFN-γ between the two groups. Immune cycle characteristics varied from one cancer type to another.

**Conclusion:** This study reveals a tumor and immune profile of HDAC pathway CNV as well as its unlimited potential in immune prognosis.

## Introduction

Histone deacetylases (HDACs) are epigenetic modifiers that are essential for structural modifications of chromosomes and regulation of gene expression, particularly gene transcriptional activity [1]. In humans, there are 18 HDAC enzymes classified as Rpd3-like proteins, Hda1-like proteins, Sir2-like proteins, and HDAC11. HDAC is subject to multiple control mechanisms, including protein interactions and post-translational modifications [2]. A recent study has shown that HDAC can promote viral pathogenicity *via* distinct mechanisms, such as activation of pro-inflammatory responses and upregulation of angiotensin-converting enzyme 2 activity [3]. Research on HDAC inhibitors (HDACi) has been the focus of immunotherapy to improve tumor recognition by immune cells and increase anti-tumor activity [4]. Given that HDAC is involved in neurodevelopment, memory formation and cognitive processes, it has been proposed that HDACi could be used as an innovative drug for the treatment of neurodegenerative diseases, such as Alzheimer’s disease [5]. Despite the fact that five HDACis, including belinostat, chidamide, panobinostat, romidepsin, and vorinostat, have been approved for clinical treatment, they are still associated with many adverse reactions, especially bone marrow suppression, diarrhea and various adverse cardiac reactions [6]. To improve the safety and efficacy of the HDACis in approved indications and to further expand their indications, the HDAC pathway in pan-cancer urgently needs to be characterized.

Copy number variation (CNV) is an important component of genomic structural variation, which is mainly manifested as deletions or duplications at the submicroscopic level. CNV has been shown to be implicated in human diseases. While CNV could help to identify the key differences between cancer and subtypes of cancer driving resistance to specific drugs, it might contribute to individualized drug therapy for patients [7]. Butler et al. showed that CNV in genes reduces the sensitivity of cancer patients to HDACi by affecting gene mRNA expression [8]. In a study of human cancers, Cohen et al. demonstrated that HDAC activity was increased in pharyngeal, renal and pancreatic cancers, while HDAC4 activation was associated with abnormalities in inflammatory and chemokine-related genes [9].

To date, studies on characterization of the HDAC pathway CNV are still lacking. To characterize the distribution and characterization of HDAC pathway CNV in pan-cancer, we performed an in-depth analysis of correlation of HDAC pathway CNV with genomic features and immune features, as well as its relationship with prognostic and immune checkpoint biomarkers.

## Materials and Methods

### Patient Information and Sample Collection

Genomic and clinical information of 10,678 pan-cancer patients were obtained from the TCGA database (https://gdc.cancer.gov/about-data/publications/panimmune) [10]. The TCGA pan-cancer cohort comprised a total of 33 different types of cancer: adrenocortical carcinoma (ACC), bladder urothelial carcinoma (BLCA), breast invasive carcinoma (BRCA), cervical squamous cell carcinoma and endocervical adenocarcinoma (CESC), cholangiocarcinoma (CHOL), colon adenocarcinoma (COAD), diffuse large B-cell lymphoma (DLBC), esophageal carcinoma (ESCA), glioblastoma (GBM), head and neck squamous cell carcinoma (HNSC), chromophobe renal cell carcinoma (KICH), renal cell carcinoma (KIRC), renal papillary cell carcinoma (KIPR), acute myeloid leukemia (LAML), low-grade brain glioma (LGG), liver hepatocellular carcinoma (LIHC), lung adenocarcinoma (LUAD), lung squamous cell carcinoma (LUSC), mesothelioma (MESO), ovarian serous cystadenocarcinoma (OV), pancreatic adenocarcinoma (PAAD), pheochromocytoma/paraganglioma (PCPG), prostate adenocarcinoma (PRAD), rectum adenocarcinoma (READ), sarcoma (SARC), skin cutaneous melanoma (SKCM), stomach adenocarcinoma (STAD), tsticular giant cell tumor (TGCT), thyroid cancer (THCA), thymoma (THYM), uterine corpus endometrial carcinoma (UCEC), and uterine carcinosarcoma (UCS). According to the pathway calculation variation, the pathway CNV was divided into three different types: CNV gain, CNV loss, and CNV gain and loss (CNA). All patients were categorized into the altered-type group or wild-type group based on the presence or absence of CNV in HDAC pathway.

### Immune Profile Analysis

In order to quantify the components of immune cells in tumor immune microenvironment (TME), we performed single-sample gene set enrichment analysis (ssGSEA) to evaluate the enrichment of 28 immune cell subsets [11, 12]. The gene sets for major histocompatibility complex class I or II molecules (MHCI/II), chemokines, cytolytic activity, IFN-γ were described in previous studies [13, 14]. R package “ESTIMATE” was used to calculate immune and stroma scores by using RNA-seq data. The cancer immune cycle refers to the anti-cancer immune response which is a dynamic process. Eight steps of the cancer immunity cycle were quantified using immunogram scores (IGSs) including: IGS1, T cell immunity; IGS2, tumor antigenicity; IGS3, priming and activation; IGS4, trafficking and infiltration; IGS5, recognition of tumor cells; IGS6, inhibitors cells; IGS7, checkpoint expression; IGS8, inhibitory molecule expression [15]. Gene Set Variation Analysis (GSVA) was conducted to assess IGSs using the “GSVA” R package.

### Molecular Features

The number of silent mutations, immunologic mutation, CNV burden scores, and loss of heterozygosity (LOH) scores were derived from published research data [10].

### Statistical Analysis

Statistical analyses were performed using R software (version 3.4.2). Differences between the two groups were analyzed using Wilcoxon rank sum test for continuous variables, and Fisher’s exact test for categorical variables. Specially, the radar charts were used to present the differences of TMB and TNB across different cancer species. The raw value of TMB and TNB was ranked in the same cancer type. After ranked, the value of TMB and TNB is between 0 and 1. The dots in radar chart represent the median percent-ranked value in two groups. Likewise, the differences of eight cancer-immunity cycle features also were exhibited by radar charts in BRCA and STAD. To test for association between copy-number and gene expression values, Spearman correlation coefficients was used. *p*-value was adjusted by FDR. Kaplan-Meier survival curves and log-rank test were used for analysis of the overall survival (OS). Cox proportional hazards model was utilized for univariate Cox regression analyses to calculate the hazard ratios (HR) with corresponding 95% confidence intervals (CI) and *p* value. *p* < 0.05 was considered statistically significant for two-sided tests.

## Results

### Histone Deacetylase Pathway Copy Number Variation in Pan-Cancerous Species

**FIGURE 1:**
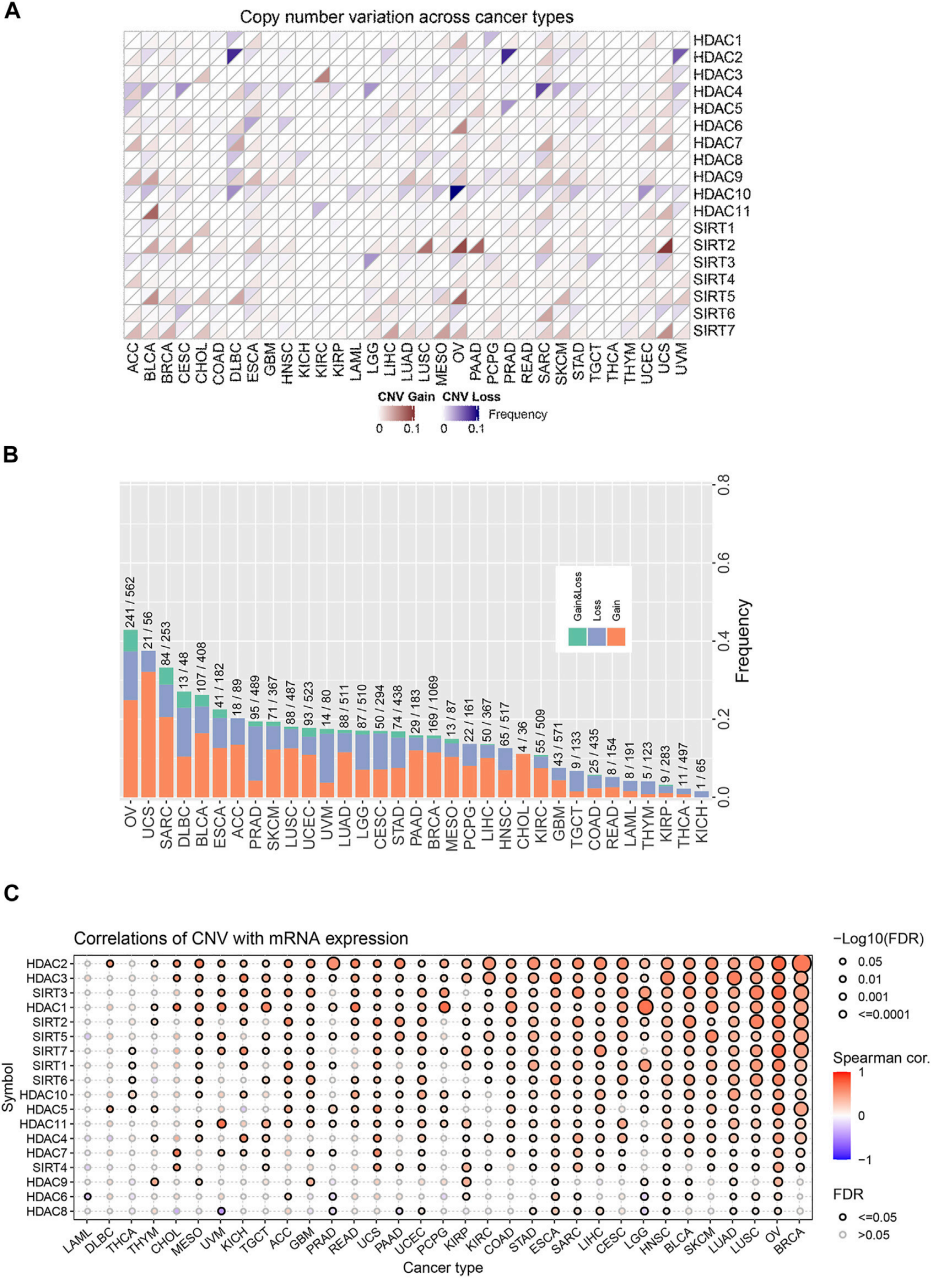
Distribution and frequency of CNV in HDAC pathway in pan-cancer. **(A)** CNV distribution of each gene in HDAC pathway in various cancer species. **(B)** Frequencies of the HDAC pathway CNV variants in various cancer species. **(C)** The correlations between CNV of HDAC gene and mRNA expression of HDAC gene in various cancer species.

**FIGURE 2:**
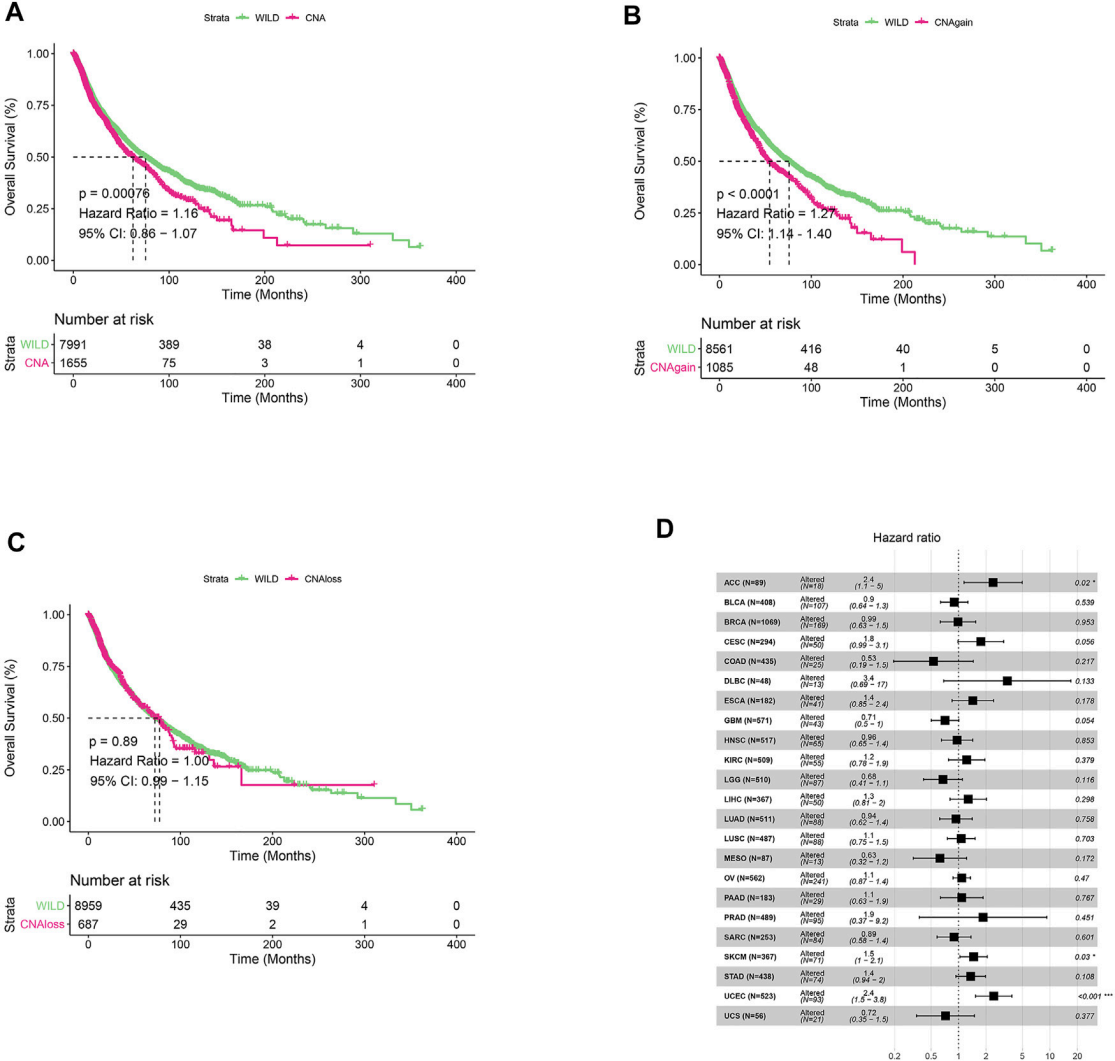
Relationship between HDAC pathway CNV and prognosis in pan-cancer. **(A)** Kaplan-Meier survival analysis of relationship between HDAC pathway CNV gain and loss (CNA) and OS in all pan-cancer species. **(B)** Relationship between HDAC pathway CNA gain and OS. **(C)** Relationship between HDAC pathway CNA loss and OS. **(D)** Univariate Cox regression analysis of relationship between pathway CNV Altered (gain and loss) and OS in various cancer types. *p < 0.05 indicates a statistically significant difference between the two groups, **p < 0.01, ***p < 0.001.

We first analyzed CNV changes in each HDAC pathway gene in the 33 types of tumors. As shown in Figures 1A,B, the distribution of CNV gain and CNV loss in HDAC pathway varied from one cancer type to another. In this case, while no CNV loss occurred in CHOL, CNV gain was absent in KICH. Moreover, there were differences in the frequency of CNV gain and CNV loss in HDAC pathway among these cancer species. Furthermore, the correlation analysis between CNV and mRNA expression in each HDAC pathway gene also performed in pan-cancers. The result was showed in Figure 1C. Blue bubbles represent negative correlations; red bubbles represent positive correlations. The deeper of the color, the higher of the correlation. Bubble size is positively correlated with the FDR significance. Black outline border indicates FDR < 0.05. The result suggested that there are significantly positive correlations between CNV and mRNA expression in each HDAC pathway gene in most cancer species. In this study, the patients with gain or loss of any HDAC genes were stratified in altered-type group, and the others were wild-type group. We further investigated the relationship between HDAC pathway CNA and OS in all pan-cancer species. As illustrated in Figures 2A,B, OS was significantly longer in the wild-type group compared with the CNA group and the CNA gain group (*p* = 0.00076 and *p* < 0.0001, respectively). Conversely, no significant correlation between the pathway CNA loss and OS was observed (Figure 2C). Besides, univariate COX regression analysis identified the HDAC pathway CNV as a risk factor for OS in cancer species including ACC, SKCM, and UCEC (Figure 2D).

### Differential Genomic Characteristics With the Occurrence of Histone Deacetylase Pathway Copy Number Variation in Pan-Cancer Species

**FIGURE 3:**
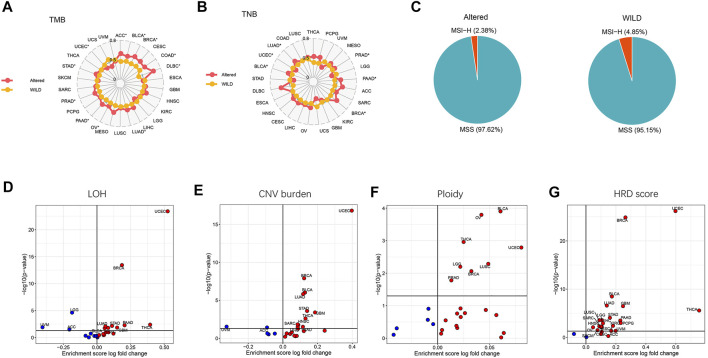
Differential tumor genomic characteristic markers in the altered-type compared with the wild-type group across pan-cancers. **(A)** Differences of percent rank-ordered TMB between the HDAC pathway CNV altered-type and wild-type groups in pan-cancer species. **(B)** Differences of percent rank-ordered TNB between the HDAC pathway CNV altered-type and wild-type groups in pan-cancer species. **(C)** The percentages of MSI-H and MSS in both CNV altered-type and wild-type groups. **(D–G)** Correlations of HDAC pathway CNV with LOH fraction, CNV burden, ploidy, HRD score. Red dots represent the corresponding value in altered-type group higher than that in the wild-type group, opposite in blue dots.

We next analyzed the differences of genomic characteristics including TMB, tumor neoantigen burden (TNB), LOH, CNV burden, ploidy, and homologous recombination defect (HRD) score across cancer types, as well as the percentages of MSI-H and MSS between the altered-type and wild-type groups. As presented in Figure 3A, compared with the wild-type group, the altered-type group displayed a significantly higher TMB in ACC, BLCA, BRCA, COAD, DLBC, LUAD, OV, PAAD, PRAD, and STAD (*p* < 0.05), but a markedly lower TMB in UCEC (*p* < 0.05). Likewise, in comparison with the wild-type group, the altered-type group had a significantly higher TNB in PRAD, PAAD, BRCA, BLCA, and LUAD (*p* < 0.05), but a markedly lower TNB in UCEC (*p* < 0.05) (Figure 3B). In the meantime, we observed that both altered-type and wild-type groups included fewer MSI-H patients and more MSS patients, while there were no significant differences in the percentages of MSI-H and MSS between the two groups (Figure 3C). Notably, with HDAC pathway CNV, there was significant difference of LOH fraction in 12 cancer types, including THCA, LGG, UVM, UCEC, GBM, PAAD, BLCA, STAD, HNSC, LUAD, ACC, and BRCA. More specifically, the LOH is significantly decreased in UVM, LGG, and ACC, while a significant increase of LOH fraction occurred in the remaining 9 cancer species (Figure 3D). Likewise, CNV burden was significantly different between the altered-type and wild-type groups in 13 cancer species, including UVM, ACC, UCEC, BRCA, LUAD, BLCA, STAD, GBM, HNSC, THCA, SARC, PAAD, and LIHC. More specifically, CNV burden was diminished in UVM and ACC and increased in other 11 cancer species (Figure 3E). Besides, ploidy was increased in eight cancer species (BLCA, OV, THCA, LGG, LUSC, UCEC, BRCA, and PRAD (*p* < 0.05) (Figure 3F), while it had a significantly improved HRD score in 21 cancer species (THCA, UVM, UCEC, KIRC, PCPG, GBM, LGG, ACC, PAAD, LIHC, STAD, SKCM, CESC, BRCA, HNSC, SARC, LUAD, BLCA, ESCA, LUSC, and OV) (*p* < 0.05) (Figure 3G).

### Effect of Histone Deacetylase Pathway Copy Number Variation on Immune Circulation Characteristics in Pan-Cancer Species

**FIGURE 4:**
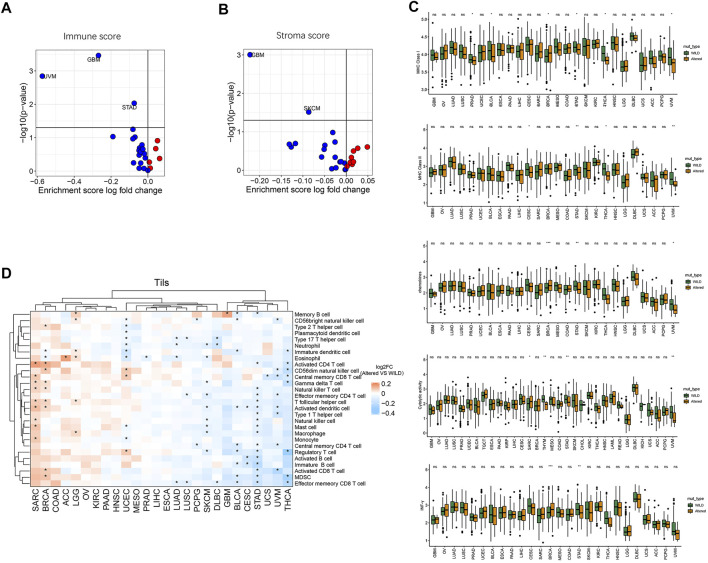
Relationship between HDAC pathway CNV and immune circulation characteristics. **(A)** Correlation between HDAC pathway CNV and immune score. **(B)** Correlation between HDAC pathway CNV and stroma score. Red dots represent the corresponding value in altered-type group higher than that in the wild-type group, opposite in blue dots. **(C)** Differences in MHC-I, MHC-II, chemokines, cytolytic-activity, and IFN-γ signature expression between the mutation and wild-type groups. **(D)** Differences in the enrichment levels of 28 immune cell subpopulations between the altered-type and wild-type groups in different cancer species and clustering analysis. *p < 0.05 indicates a statistically significant difference between the two groups.

We further analyzed differences in immune cycle characteristics including major histocompatibility complex class I (MHC-I), MHC class II (MHC-II), chemokines, cytolytic-activity, IFN-γ, immune score, and stroma score among the cancer species as well as the differences between the two groups. As shown in Figures 4A,B, with the pathway CNV alterations, there was a significant decrease of immune score in cancer species GBM, UVM, and STAD (*p* < 0.05), the stroma score was significantly decreased in GBM and SKCM (*p* < 0.05). Compared with the wild-type group, the altered-type group had a significantly lower level of MHC-I in PRAD, BLCA, STAD, THCA, and UVM (*p* < 0.05), but a markedly higher level of MHC-I in BRCA (Figure 4C). Moreover, the altered-type group exhibited a significantly lower level of MHC-II in CESC, STAD, THCA, and UVM, a significantly higher level of chemokine in BRCA, and a lower level of chemokine in STAD and UVM than the wild-type group (*p* < 0.05) (Figure 4C). Besides, the altered-type group had a significantly higher level of cytolytic activity than the wild-type group in SARC, while the opposite results were found in STAD and UVM (*p* < 0.05). In comparison with the wild-type group, the altered-type group displayed a significantly higher level of IFN-γ in BRCA, but a markedly lower level of IFN-γ in STAD (*p* < 0.05).

Moreover, we performed clustering analysis of cancer types and immune cell subgroups. As illustrated in Figure 4D, 28 immune cell subgroups were more enriched in the altered-type group compared with the wild-type group in cancer species SARC, BRCA, COAD, ACC, LGG, OV, KIRC, PAAD, and HNSC, while they were more enriched in the wild-type group in GBM, BLCA, CESC, STAD, UCS, UVM, and THCA. While eosinophil was significantly more enriched in the altered-type group as compared to the wild-type group in ACC cancer type, neutrophil, eosinophil, activated dendritic cell, natural killer cell, macrophage, and regulatory T cell were significantly more enriched in the wild-type group in SKCM. In UCEC, neutrophil and eosinophil were significantly more enriched in the wild-type group compared with the altered-type group, whereas a markedly more enrichment of CD56dim natural killer cell, central memory CD8 T cell and regulatory T cell was detected in the altered-type group.

### The Relationship Between Histone Deacetylase Pathway Copy Number Variation and Immune Checkpoint Biomarkers in Pan-Cancer Species

**FIGURE 5:**
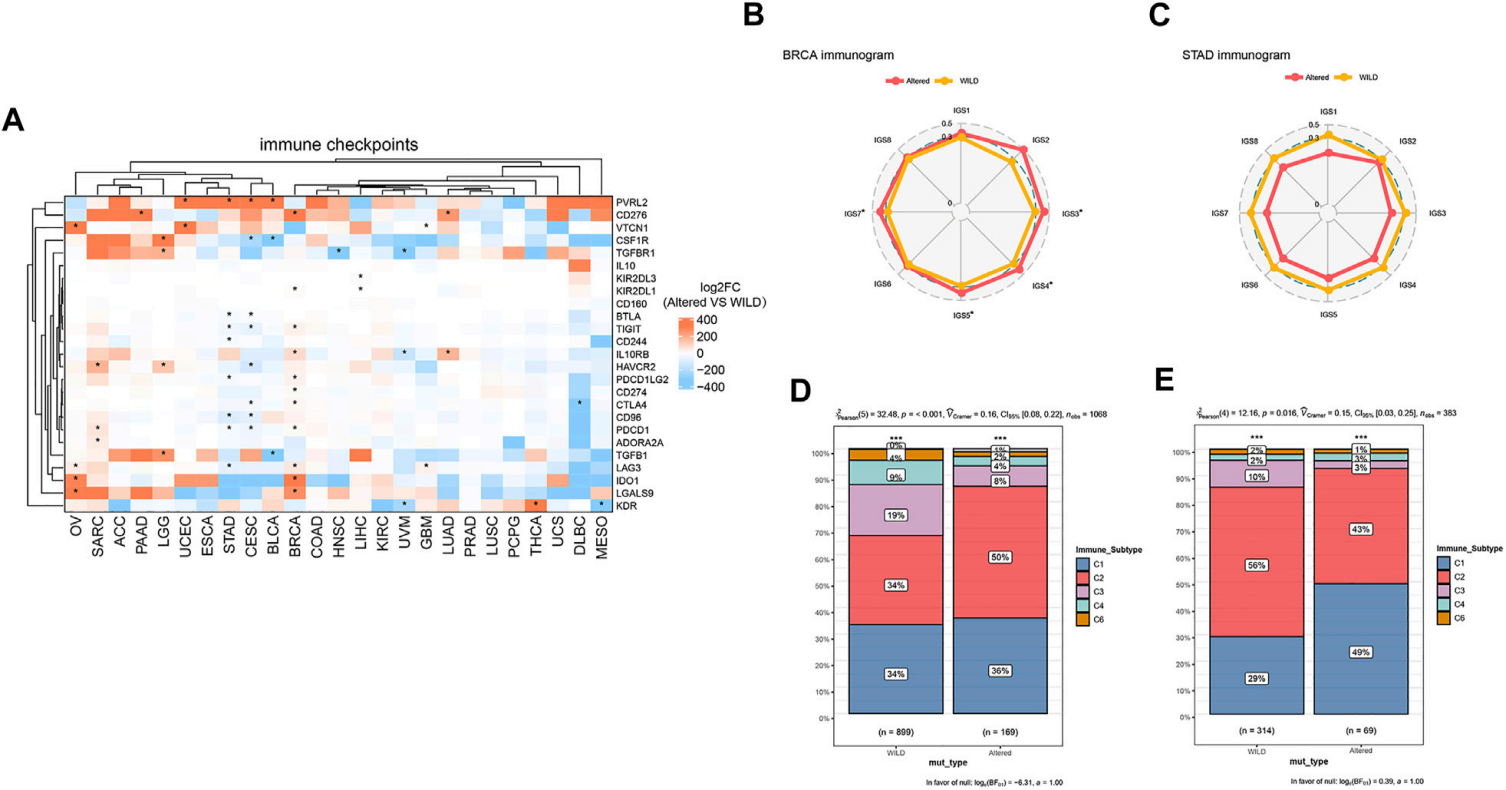
Relationship between HDAC pathway CNV and immune checkpoints. **(A)** Differentially expressed immune checkpoints in the HDAC pathway CNV altered-type group across pan-cancer species. **(B,C)** Differences of percent rank-ordered immune cycle between the HDAC pathway CNV altered-type and wild-type groups in BRCA and STAD. **(D,E)** Composition ratio analysis of different immune subtypes of HDAC pathway CNV in BRCA and STAD. *p < 0.05 indicates a statistically significant difference between the two groups.

Finally, we performed cluster analysis of cancer types and immune checkpoint biomarkers, and comparatively analyzed differential enrichments of the biomarkers between the two groups. As shown in Figure 5A, there was no significant correlation between the CNV and immune checkpoints in cancer species ACC, ESCA, COAD, KIRC, PRAD, LUSC, PCPG, and UCS, while PVRL2 and VTCN1 were significantly higher expressed in the altered-type group compared with the wild-type group in UCEC. Analysis of cancer-immunity cycle (CIC) features of HDAC pathway CNV revealed a significant difference in immune cycle stages IGS3, IGS4, IGS5, and IGS7 between the two groups in BRCA (*p* < 0.05) (Figure 5B). On the contrary, there were no significant differences in all 8 immune cycle stages between the two groups in STAD (*p* > 0.05) (Figure 5C). In BRCA and STAD, statistically significant differences in composition ratios of the different immune subtypes (C1–C6) were identified between the two groups (BRCA, *p* < 0.001; STAD, *p* = 0.016) (Figures 5D,E).

## Discussion

In this study, we performed a comprehensive genomic and immunophenotypic analysis of the HADC pathway CNV in 10,678 pan-cancer patients across 33 cancer types from the latest chemotherapy cohort in the TCGA database. The present study identified both HDAC pathway CNV and CNV gain as prognostic risk factors for pan-cancer types. More specifically, HDAC pathway CNV was found to be an independent risk factor for poor prognosis in patients with ACC, SKCM, and UCEC. Previous studies have shown that CNV and HDAC are implicated in the development and treatment of ACC, SKCM, and UCEC [16–18]. Here, we provided the first demonstration that HDAC pathway CNV can serve as a potential biomarker for poor prognosis of cancer species ACC, SKCM, and UCEC.

To further characterize HDAC alterations, all samples in this study were divided into the altered-type and wild-type groups based on the presence or absence of CNV in the HDAC pathway. Notably, we observed that tumor genomic characteristics including TMB, TNB, MSI-H, and MSS in the altered-type group varied from one cancer type to another. MSI and TMB are mutually independent biomarkers that complement each other to predict the efficacy of immune checkpoint inhibitors (ICIs). It has been shown that combination of MSI and TMB information can differentiate among cancer patients with distinct microenvironments [19]. Consistently, the present study demonstrated that TMB was significantly associated with HDAC pathway CNV in most cancers. Similarly, Liu et al. suggested a possible role of combined TMB and CNA in distinguishing one metastatic tumor from another with a different prognosis and clinical response to ICI treatment [20]. Furthermore, patients with TMB-H and/or MSI-H may preferentially benefit from ICI treatment [21], and frequent HDAC2 mutations have been identified in MSI tumors [22]. Here, we showed that fewer patients with MSI-H and more patients with MSS in the HDAC pathway altered-type group. Sefrioui et al. found that CNV was significantly associated with disease-free survival in stage II–III colon cancer with MSS [23]. The present study provided more evidence suggesting that HDAC pathway CNV combined with MSS index may serve as a prognostic indicator for ICI treatment in cancer patients. It has been reported that prognosis of cancer patients can largely be predicted alternatively by detecting tumor cytogenetic aberrations such as single-nucleotide polymorphisms, CNV and LOH [24]. In this study, we showed that HDAC pathway CNV was positively correlated with immune escape related genomic characteristics including LOH, CNV burden, ploidy, and HRD score in most cancer species.

In addition to genomic features, this study investigated immune signature indicators in human cancers. We found that HDAC pathway CNV was significantly enriched in some cancer species, while it was negatively correlated with immune score and stroma score. These findings are consistent with a previous study revealing a negative correlation between immune score and CNV in pancreatic cancer with a good prognosis [25]. Gene expression dysregulation caused by copy number changes has been shown to be significantly related to the immune infiltration and survival [26]. Meanwhile, regulation of HDAC levels may lead to improvement of clinical efficacy of HDACi in chemotherapy [27]. In the present study, we further analyzed the immune circulation profile and observed that there were significant differences in the levels of MHC-I, MHC-II, chemokines, cytolytic-activity, and IFN-γ between HDAC pathway CNV altered-type group and the wild-type group. Inhibition of HDAC pathway facilitates restoration of DNA damage-dependent MHC-I expression in cancer cells, resulting in immune activation to enhance antitumor effects [28]. Neuwelt et al. showed that HDAC inhibition enhances the expression of MHC II to a certain extent [29]. Chemokines and their receptors are capable of controlling the migration and residence of all immune cells [30]. In a recent study, Zheng et al. showed that HDACi can induce the expression of T cell chemokines in tumors, thus enhancing the response to immunotherapy [31]. Here, we demonstrated that HDAC pathway CNV was significantly associated with chemokines in SARC, BRCA, STAD, and UVM, suggesting that combination of HDACi and immunotherapy may potentially be of significance in the cancer treatment. These results can be justified by the observation that HDACi exerts an enhancing effect on IFN-γ expression and is markedly correlated with IFN-γ [32].

In conclusion, this study shows that dysregulation of the HDAC pathway CNV can affect the expression of various immune cell subpopulations and immune checkpoints in the TME, indicating an important role of the CNV in cancer immunotherapy. Thus, HDAC pathway CNV may become a focus of interest in study of combined HDACi and immunotherapy as a promising potential prognostic indicator.

## Data Availability

Publicly available datasets were analyzed in this study. This data can be found here: TCGA database (https://gdc.cancer.gov/about-data/publications/panimmune).

## Glossary

ACC: adrenocortical carcinoma
BLCA: bladder urothelial carcinoma
BRCA: breast invasive carcinoma
CAN: CNV gain and loss
CESC: cervical squamous cell carcinoma and endocervical adenocarcinoma
CHOL: cholangiocarcinoma
CNV: copy number variation
COAD: colon adenocarcinoma
DLBC: diffuse large B-cell lymphoma
ESCA: esophageal carcinoma
GBM: glioblastoma
GSVA: gene set variation analysis
HDACi: HDAC inhibitors
HDACs: Histone deacetylases
HNSC: head and neck squamous cell carcinoma
HRD: homologous recombination defect
ICIs: immune checkpoint inhibitors.
IGSs: immunogram scores
KICH: chromophobe renal cell carcinoma
KIPR: renal papillary cell carcinoma
KIRC: renal cell carcinoma
LAML: acute myeloid leukemia
LGG: low-grade brain glioma
LIHC: liver hepatocellular carcinoma
LUAD: lung adenocarcinoma
LUSC: lung squamous cell carcinoma
MESO: mesothelioma
MHC-I: major histocompatibility complex class I
MHC-II: MHC class II
OS: overall survival
OV: ovarian serous cystadenocarcinoma
PAAD: pancreatic adenocarcinoma
PCPG: pheochromocytoma/paraganglioma
PRAD: prostate adenocarcinoma
READ: rectum adenocarcinoma
SARC: sarcoma
SKCM: skin cutaneous melanoma
STAD: stomach adenocarcinoma
TGCT: tsticular germ cell tumor
THCA: thyroid carcinoma
THYM: thymoma
TME: tumor immune microenvironment
TNB: tumor neoantigen burden
UCEC: uterine corpus endometrial carcinoma
UCS: uterine carcinosarcoma
